# Supplementary material for: ERK Inhibitor Ulixertinib Inhibits High-Risk Neuroblastoma Growth In Vitro and In Vivo
Source: Cancers (Basel). 2022 Nov 10;14(22):5534. doi: 10.3390/cancers14225534 (PMC9688897; doi:10.3390/cancers14225534)
Supplement: Supplementary file 1 [file cancers-14-05534-s001.zip › Figure-S3.pdf]

A

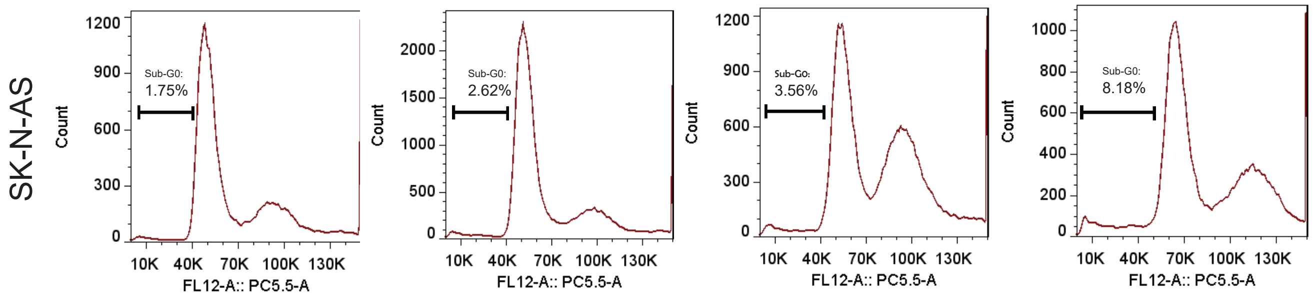

Ulixer (0.5 μM, 24h):	-	+	-	+
Dox (0.5 μM, 24h):	-	-	+	+

B

SK-N-AS (sub-G0)				
	Control (%)	Ulixertinib (%)	Doxorubicin (%)	U+D (%)
1	1.75	2.62	3.56	8.18
2	2.04	2.40	3.81	7.82
3	1.62	2.24	3.03	8.04
Aver±SD	1.8±0.22	2.42±0.19	3.46±0.40	8.02±0.18

C

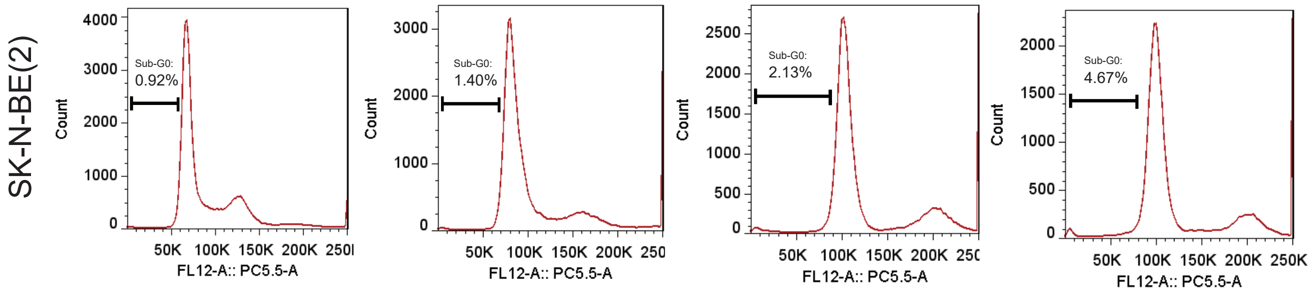

Ulixer (0.5 μM, 24h):	-	+	-	+
Dox (0.5 μM, 24h):	-	-	+	+

D

SK-N-BE(2) (sub-G0)				
	Control (%)	Ulixertinib (%)	Doxorubicin (%)	U+D (%)
1	0.92	1.40	2.13	4.67
2	1.12	1.66	2.60	4.18
3	0.67	1.36	1.77	4.25
Aver±SD	0.90±0.23	1.48±0.16	2.17±0.42	4.37±0.27

Figure S3
