# Supplementary material for: ERK Inhibitor Ulixertinib Inhibits High-Risk Neuroblastoma Growth In Vitro and In Vivo
Source: Cancers (Basel). 2022 Nov 10;14(22):5534. doi: 10.3390/cancers14225534 (PMC9688897; doi:10.3390/cancers14225534)
Supplement: Supplementary file 1 [file cancers-14-05534-s001.zip › Supplementary Figure&table legend-F1R.pdf]

Figure legend

Figure S1. High expression of targets induced by ulixertinib treatment in NB cells correlates with a better NB patient survival. Correlations of MBNL2 (A), SEPT4 (B), REPS2 (C), OTUD5 (D), TXNIP (E), and PRUNE2 (F) mRNA levels in NB tissues with the overall survival of NB patients were analyzed using the GSE62564 (Seqc-498-cohort) dataset (http://r2.amc.nl).

Figure S2. High expression of targets inhibited by ulixertinib treatment in NB cells correlates with a poor NB patient survival. Correlations of CDCA5 (A), ATAD2 (B), CDC25A (C), CDK2 (D), ANAPC10 (E), RRM2 (F), LGR5 (G), SKP2 (H), CENPM (I), and UBE2T (J) mRNA levels in NB tissues with the overall survival of NB patients were analyzed using the GSE62564 (Seqc-498-cohort) dataset (http://r2.amc.nl).

Figure S3. Ulixertinib sensitizes human NB cells to Doxorubicin-induced cell apoptosis. Human NB cell lines, SK-N-AS (A) and SK-N-BE(2) (C) were treated with ulixertinib (0.5 μM) or Doxorubicin (0.5 μM) or their combination for 24 h and examined by flow cytometry using PI staining to label apoptotic cells (sub-G0 DNA contents). (A, C) Representative histograms of PI staining analyses in ulixertinib, Doxorubicin, and their combination-treated SK-N-AS (A) and SK-N-BE(2) (C) cells. (B, D) The percentages of sub-G0 contents of SK-N-AS (B) and SK-N-BE(2) (D) cells treated with ulixertinib or Doxorubicin or their combination. All results are expressed as mean ± SD values from three independent experiments.

Table legend

Table S1. Differential gene expression (DGE) analysis of RNA-Seq.

Table S2. Differential gene expression (DGE) analysis of proteins from MS.
