# Supplementary figures and images for: ERK Inhibitor Ulixertinib Inhibits High-Risk Neuroblastoma Growth In Vitro and In Vivo
Source: Cancers (Basel). 2022 Nov 10;14(22):5534. doi: 10.3390/cancers14225534 (PMC9688897; doi:10.3390/cancers14225534)

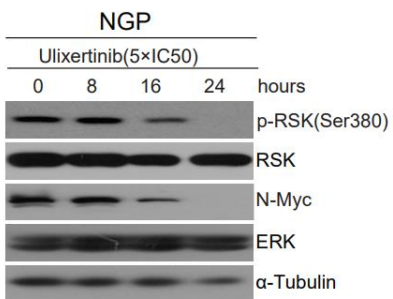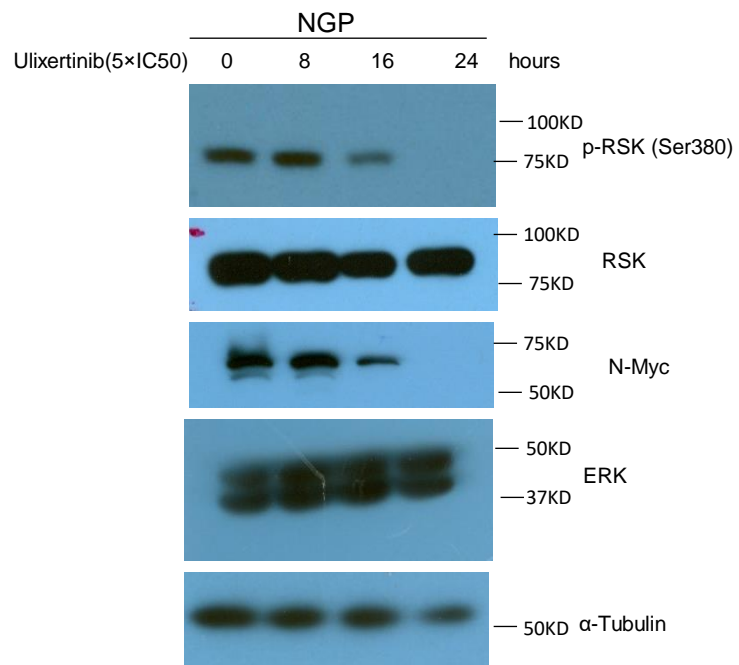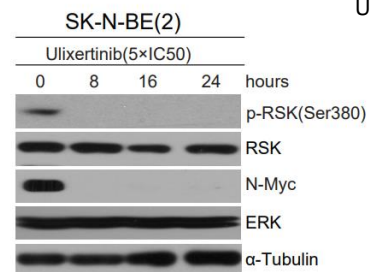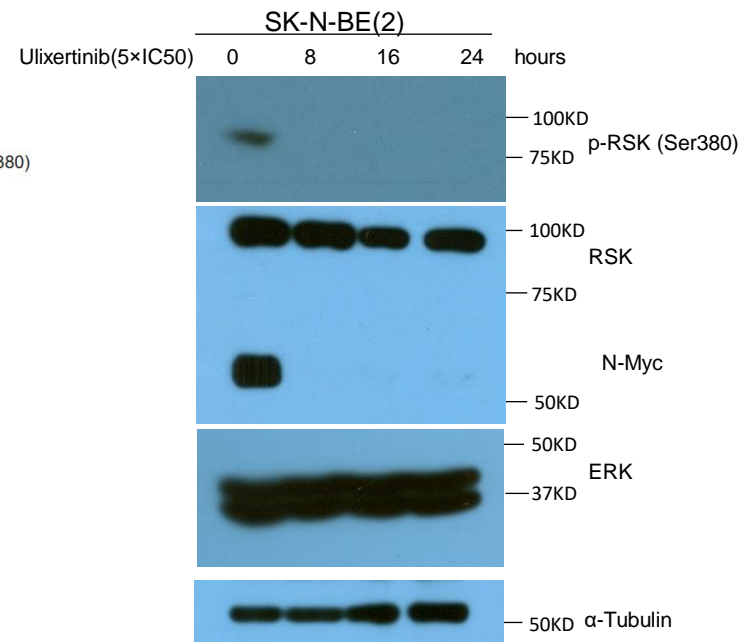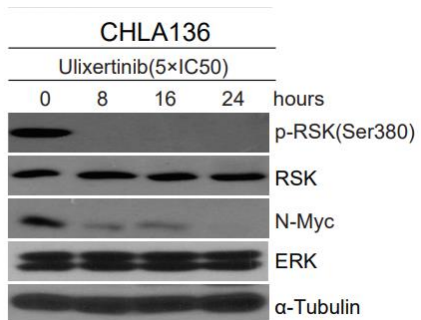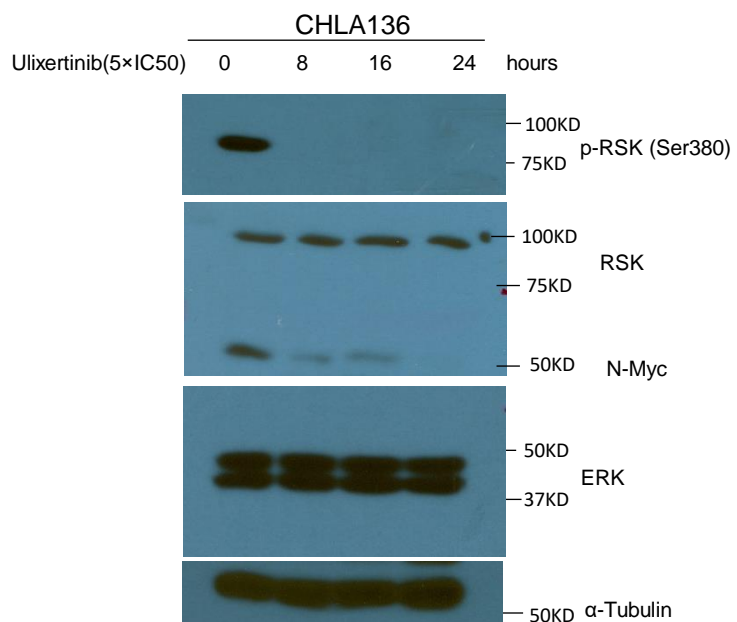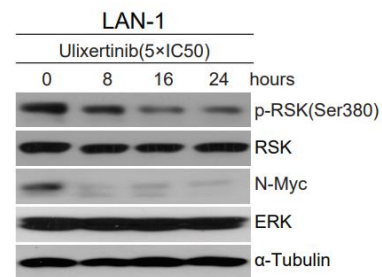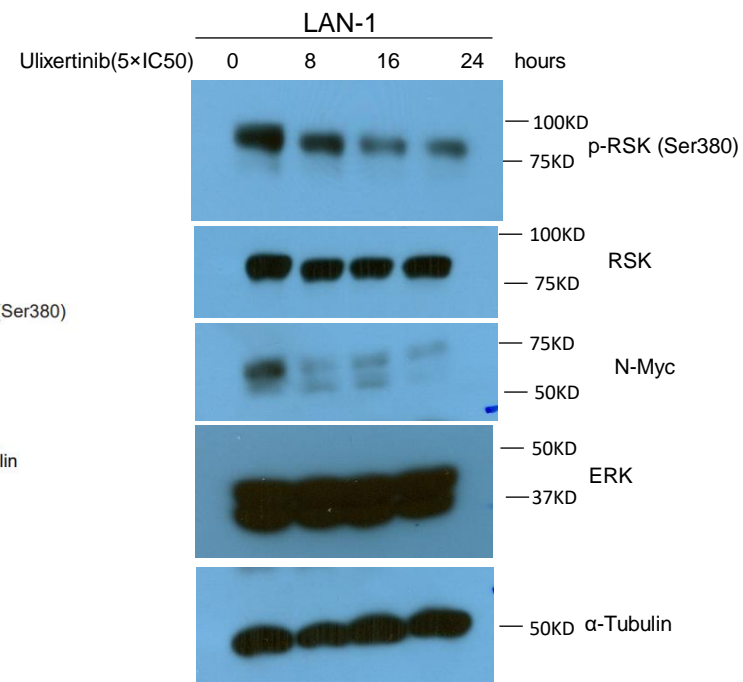

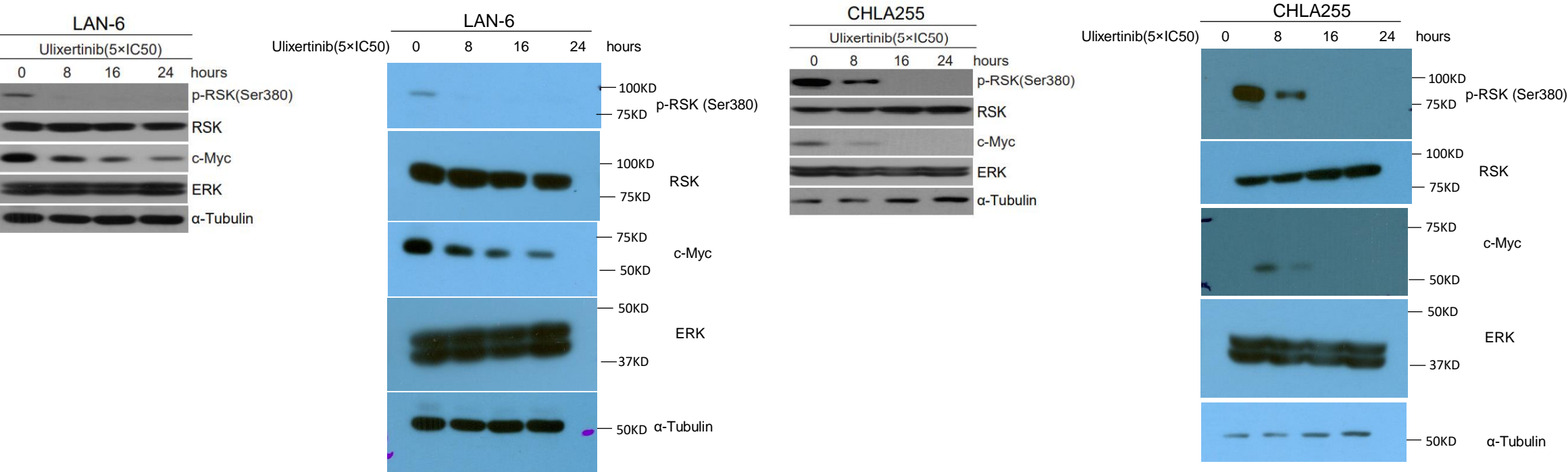

Figure. 2

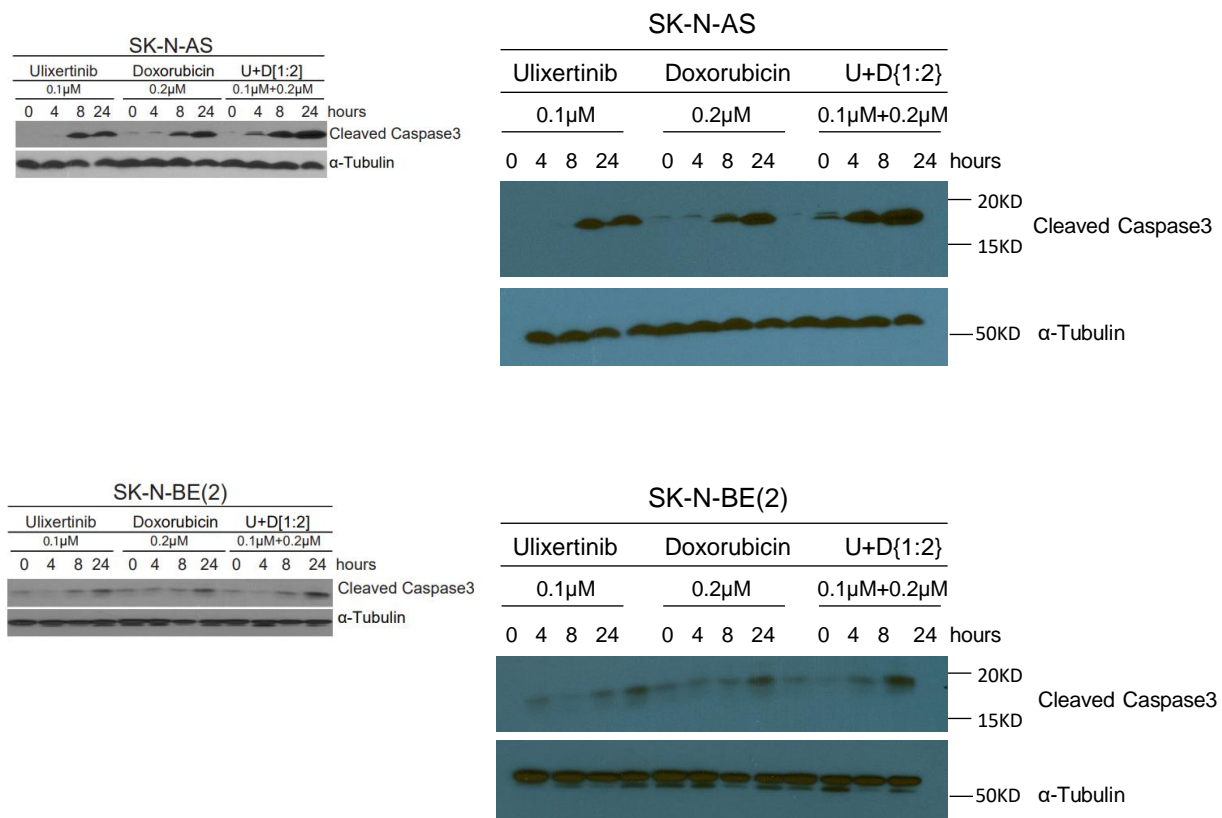

Figure. 4

Supplement: Supplementary file 1 [file cancers-14-05534-s001.zip › cancers-1968771- original westernblot.pdf]

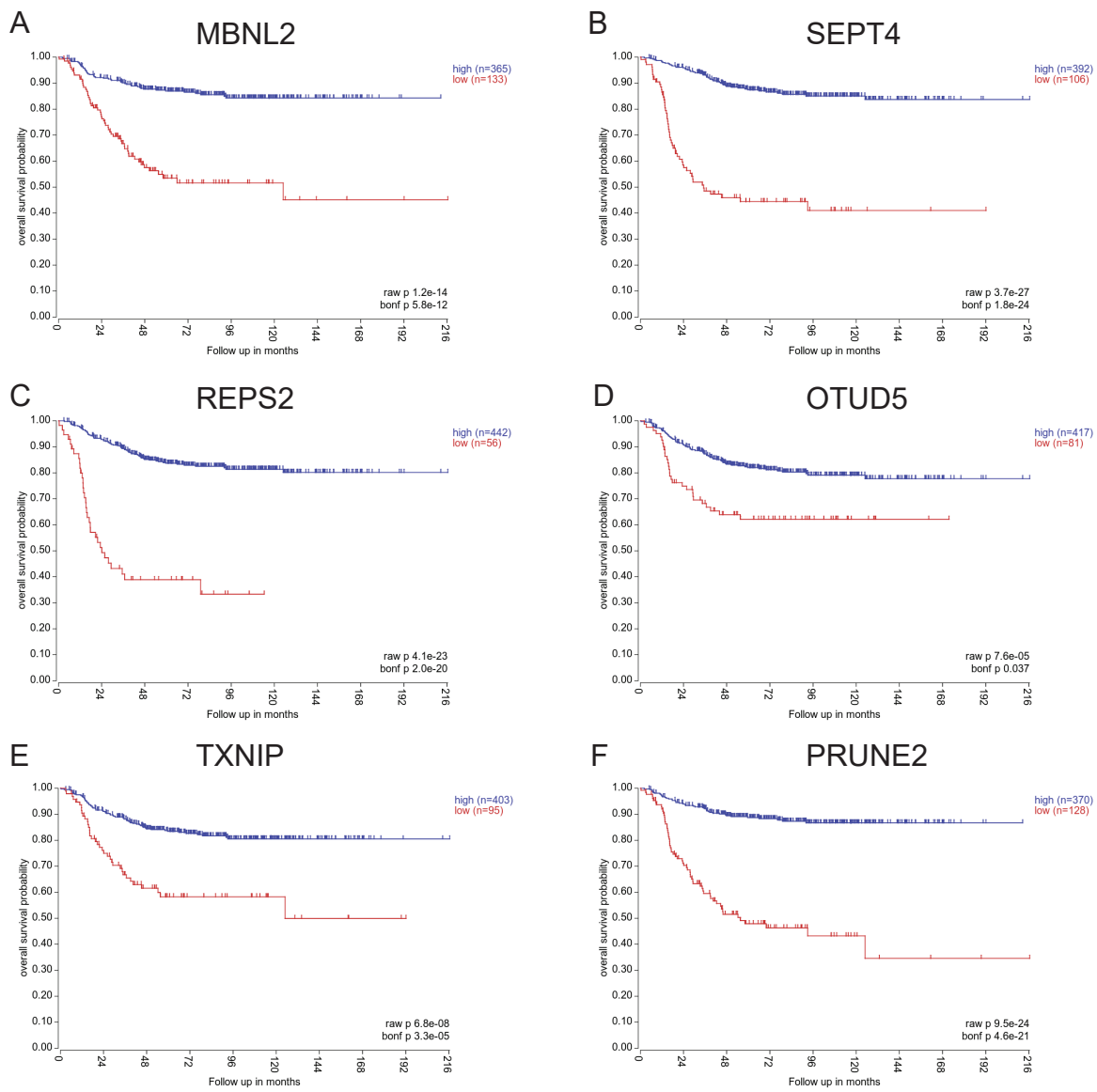

Figure S1

Supplement: Supplementary file 1 [file cancers-14-05534-s001.zip › Figure-S1FR.pdf]

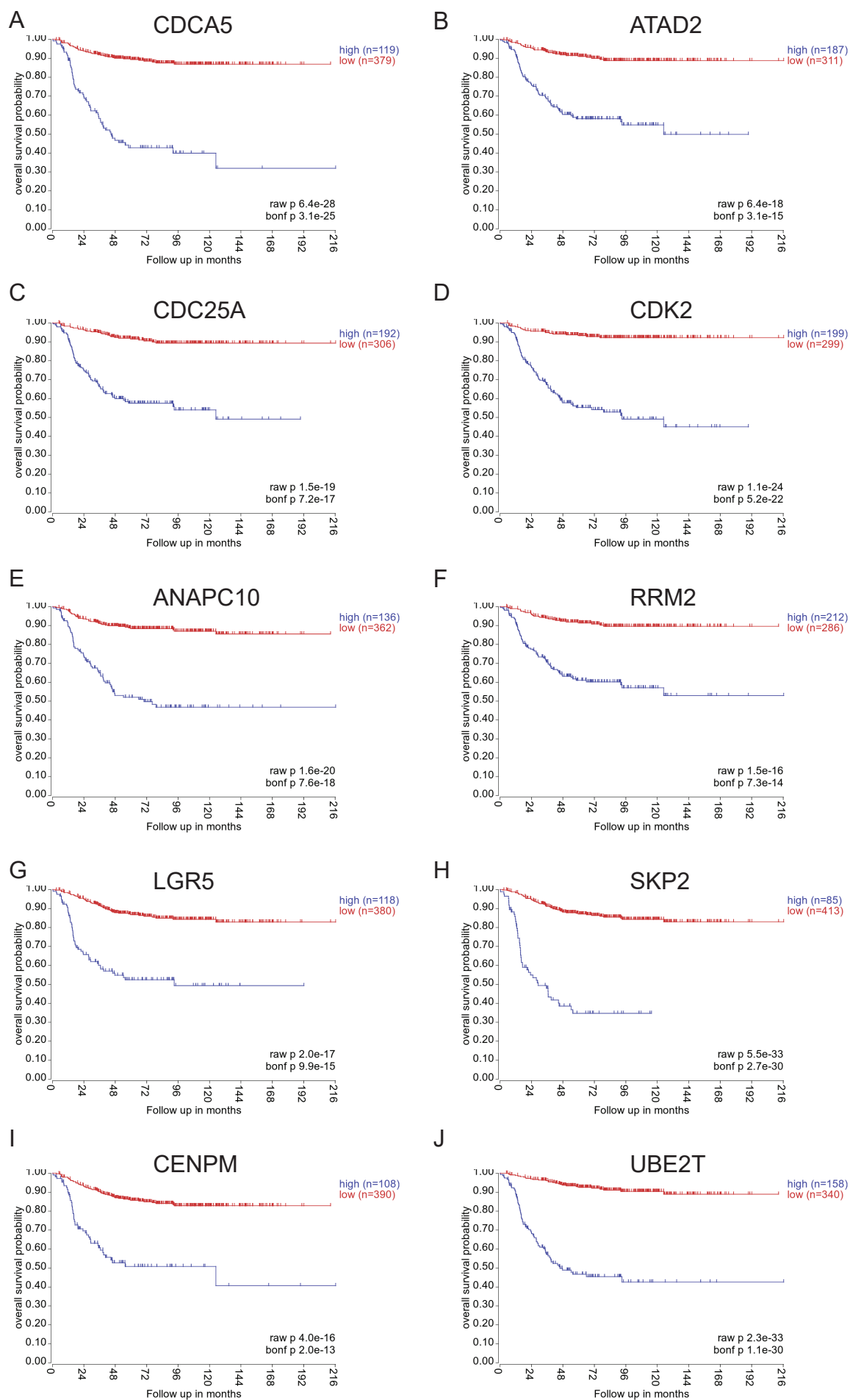

Figure S2

Supplement: Supplementary file 1 [file cancers-14-05534-s001.zip › Figure-S2FR.pdf]
